# Susceptibility of Cell Lines from Different Species to Porcine Deltacoronavirus

**DOI:** 10.3390/v18070776

**Published:** 2026-07-15

**Authors:** Hui Jiang, Baoguo Lu, Yimin Chu, Mingyue Ma, Nannan Zhang, Yanyan Xu, Yanying Chen, Qingyang Xiao, Ting Wang, Qi Peng

**Affiliations:** 1 College of Animal Science and Technology, Jiangxi Agricultural University, Nanchang 330045, China; 2 Institute of Pathogenic Microorganism, Jiangxi Agricultural University, Nanchang 330045, China; 3 College of Bioscience and Engineering, Jiangxi Agricultural University, Nanchang 330045, China; 4 School of Medical and Information Engineering, Gannan Medical University, Ganzhou 341000, China

**Keywords:** porcine deltacoronavirus, cell line susceptibility, trypsin, aminopeptidase N

## Abstract

Porcine deltacoronavirus (PDCoV) is an emerging swine enteric coronavirus that causes severe diarrhea in piglets, leading to extensive economic losses in the global swine industry. Previous studies have reported that PDCoV can experimentally infect multiple animal species. However, the replication capability of PDCoV in non-natural host cell lines remains largely unknown. Herein, we investigated the effects of exogenous trypsin on PDCoV replication and systematically evaluated the susceptibility of various cell lines derived from human, murine, and feline to PDCoV. The results showed that highly permissive cell lines (H1299, Caco-2, MC-38) supported efficient viral replication without the requirement for exogenous trypsin, and high concentrations of trypsin induced prominent syncytia formation in infected H1299, U251, and Neuro-2a cells. Furthermore, this study also found that trypsin can enhance PDCoV replication in a concentration-dependent manner in semi-permissive cell lines. PDCoV can replicate in multiple cell lines of human, murine, and feline origin. Human cells (H1299, Caco-2) and murine MC-38 are highly susceptible to PDCoV infection, whereas MCF-7, TE-10, and KYSE-410 cells were completely non-permissive. The results also revealed a potential correlation between basal APN expression and cellular susceptibility to PDCoV. Collectively, this study provides a basis for assessing the interspecies transmission and zoonotic risk of PDCoV.

## 1. Introduction

Porcine deltacoronavirus (PDCoV) is an emerging swine enteric coronavirus belonging to the genus *Deltacoronavirus* within the family *Coronaviridae*. PDCoV can infect pigs of all ages, especially piglets under one week old, which induces severe diarrhea and dehydration, consequently causing substantial economic losses to the swine industry [1]. PDCoV was first identified in 2012 during a molecular surveillance study in Hong Kong and subsequently caused an outbreak in the United States in 2014 [2,3]. In 2015, an epidemiological investigation of PDCoV conducted by Chinese researchers reported a positive rate of 33.71% for PDCoV infection in China [4]. Since then, PDCoV has been detected in many pig-raising countries, including South Korea [5], Japan [6], Thailand [7], Canada [8], Vietnam [9], Laos [10], Peru [11], and Mexico [12]. In addition to swine hosts, PDCoV has been confirmed to experimentally infect multiple vertebrate species, including wild birds [13], ferrets [14], chickens [15], turkeys [16], calves [17], and mice [18]. A critical study further identified PDCoV in plasma of three pediatric patients with acute febrile illness, providing direct evidence of the potential cross-species and zoonotic transmission of PDCoV to humans [19].

PDCoV is a single-stranded, positive-sense RNA virus with a genome of approximately 25 kb in length, encoding four structural proteins (nucleocapsid [N], membrane [M], envelope [E], and spike [S] proteins), 15 nonstructural proteins (nsp2 to nsp16), and three accessory proteins (NS6, NS7, and NS7a) [20]. The E, M, and N genes are highly conserved and often serve as target genes for the design of molecular detection assays [21,22,23]. The S protein is a type I transmembrane glycoprotein comprising an N-terminal S1 subunit and a C-terminal S2 subunit, the latter of which determines the host range of viral infection [24]. The S1 subunit is essential for binding to host receptors and initiates the early stage of infection [25]; the S2 subunit mediates fusion of the viral membrane with the host cell membrane [26]. Previous studies have shown that the PDCoV S protein engages the aminopeptidase N (APN) for entry into host cells, and that overexpression of APN can promote PDCoV replication in non-susceptible cells [27,28]. After binding to the cellular receptor, the S1 subunit will undergo a conformational rearrangement that exposes the S1/S2 cleavage site to proteases [29,30]. The S protein is proteolytically cleaved into S1 and S2 subunits, and this cleavage event is critical for mediating membrane fusion [31,32]. Consequently, exogenous proteases can facilitate the efficient replication of certain CoVs in certain cell lines, including porcine epidemic diarrhea virus (PEDV) [33], swine acute diarrhea syndrome coronavirus (SADS-CoV) [34], and PDCoV [35].

Many CoVs can cross the species barrier to diverse animal hosts, accompanied by subsequent host adaptation, and some can even establish infections in humans. Representative examples include severe acute respiratory syndrome coronavirus (SARS-CoV) and Middle East respiratory syndrome coronavirus (MERS-CoV), which were transmitted to humans from civets and camels, respectively [36,37]. PDCoV has also been documented to possess cross-species transmission potential. Accordingly, more information on PDCoV infection is urgently needed, and it is critical to evaluate its potential to replicate in cell lines derived from non-natural host species. In this study, we demonstrate that PDCoV displays an exceptionally broad species tropism in vitro and can infect cell lines from murine, feline, and human. Additionally, we show that exogenous trypsin enhances PDCoV replication and syncytium formation in some cell lines. Collectively, these findings provide a foundation for elucidating the interspecies transmission risk and host range of PDCoV.

## 2. Materials and Methods

### 2.1. Cell Culture, Virus, and Antibodies

LLC-PK1, H1299, H460, HepG2, U251, MCF-7, TE-10, KYSE-30, KYSE-410, CT26, MC-38, F81, and CRFK cells were stored in our laboratory and cultured in DMEM containing 10% fetal bovine serum (FBS, Transgene Biotech, Beijing, China) at 37 °C and 5% CO_2_. Caco-2 and Neuro-2a cells were kindly provided by Dr. Ji Cao and Dr. Sha Li from Jiangxi Agricultural University and cultured in DMEM supplemented with 10% FBS (Transgene Biotech, Beijing, China) at 37 °C and 5% CO_2_, respectively. PDCoV strain CH/JXJGS01/2016 (GenBank accession No. KY293677) was isolated in Jiangxi in 2016 and propagated in LLC-PK1 cells supplemented with 5 µg/mL trypsin at 37 °C with 5% CO_2_. Mouse anti-PDCoV N monoclonal antibody was generated in our laboratory. HRP-conjugated goat anti-mouse IgG (Cat. No. AS003) and HRP-conjugated goat anti-rabbit IgG (Cat. No. AS014) were purchased from Abclonal (Wuhan, China). Alexa Fluor 488- conjugated secondary antibody (Cat. No. A10235) was purchased from Thermo Fisher Scientific (Waltham, MA, USA).

### 2.2. Virus Infection

Cells of different species were seeded onto 24-well plates and cultured in a humidified atmosphere at 37 °C with 5% CO_2_. When the cells were grown to monolayers, cells were infected with PDCoV at an MOI of 0.1. After 1 h of infection at 37 °C, the cells were washed with phosphate-buffered saline (PBS) for two times to remove the unbound viruses. Subsequently, the cells were cultured in DMEM supplemented with 2 μg/mL trypsin at 37 °C with 5% CO_2_.

### 2.3. Immunofluorescence Assay (IFA)

Cells from human, murine, and feline were seeded into 24-well plates and cultured in a humidified atmosphere at 37 °C with 5% CO_2_. When the cells reached 100% confluency, the cells were infected with PDCoV at an MOI of 0.1. After 1 h of incubation, the cells were washed with phosphate-buffered saline (PBS) for two times to remove unbound viruses. Subsequently, the infected cells were cultured for the indicated time points (detailed time points are provided in the corresponding figure legends) in DMEM supplemented with 2 μg/mL trypsin at 37 °C with 5% CO_2_. The cells were then fixed with 4% paraformaldehyde for 15 min at room temperature (RT). After washing three times with PBS, the cells were permeabilized with 0.2% Triton X-100 for 20 min, followed by washing thrice with PBS and blocked with 5% skimmed milk for 1 h at 37 °C. Cells were rinsed with PBS three times, then incubated with mouse anti-PDCoV nucleocapsid monoclonal antibody at 1:500 dilution for 1 h at 37 °C, followed by incubation with Alexa Fluor 488- conjugated secondary antibody at 1:1000 dilution for 1 h at 37 °C. Finally, cells were stained with 0.01% 4′,6-diamidino-2-phenylindole (DAPI) and washed three times. Fluorescent images were generated with a fluorescence microscope (Nikon, Tokyo, Japan).

### 2.4. Western Blotting Analysis

Cells were harvested and lysed in NP-40 lysis buffer containing protease inhibitor PMSF (Beyotime, Shanghai, China). The cell lysates were denatured at 100 °C for 10 min and separated by sodium dodecyl sulfate–polyacrylamide gel electrophoresis. After electroblotting onto polyvinylidene difluoride membranes (Millipore, Billerica, MA, USA), the membranes were blocked with 5% skimmed milk, and then probed with indicated primary and secondary antibodies and visualized using the chemiluminescent substrate. Protein bands were visualized using an enhanced chemiluminescence (ECL) solution (Cat. No. SB-WB012, Share Bio, Shanghai, China).

### 2.5. Quantitative Real-Time Reverse Transcription PCR

Total RNA was extracted from cells of different species using TRIzol reagent (Cat. No. 9108, TaKaRa, Dalian, China) according to the manufacturer’s instructions. First-strand cDNA was synthesized with a First-Strand cDNA Synthesis Kit (Cat. No. AT311, TransGen Biotech, Beijing, China). Quantitative real-time PCR (qPCR) was performed in a 20 μL reaction mixture containing 10 μL Universal SYBR qPCR Master Mix (Cat. No. Q711, Vazyme Biotech, Nanjing, China), 0.5 μL cDNA template, and 10 μM forward and reverse primers. The thermal cycling conditions were set as follows: 95 °C for 5 min, followed by 40 cycles of 95 °C for 10 s and 60 °C for 30 s. All reactions were run in triplicate using the ABI 7500 Real-Time PCR System (Applied Biosystems, Carlsbad, CA, USA). Viral load was determined by absolute quantification qPCR with the pCAGGS-HA-PDCoV N plasmid as the standard. Relative mRNA expression levels of the APN gene were calculated using the 2^(−ΔΔCt)^ method and normalized to β-actin. The primer sequences used for RT-qPCR are listed in Supplementary Table S1.

### 2.6. Viral Titer Determination

Viral titers were determined using the 50% tissue culture infective dose (TCID_50_) assay. LLC-PK1 cells were seeded into 96-well plates and cultured in DMEM supplemented with 10% FBS (Transgene Biotech). Once cells reached full confluence, they were washed three times with PBS and inoculated with 100 μL of 10-fold serially diluted samples. Following incubation at 37 °C for 1 h, the inocula were removed, and cells were washed once with PBS before the addition of 1 mL of DMEM containing 5 μg/mL trypsin. Viral titers were subsequently calculated according to the Reed–Muench method.

### 2.7. Phylogenetic Analysis

To investigate sequence divergence of *APN* across diverse species and its potential correlation with host cell tropism, the nucleotide sequences of *APN* from 22 representative vertebrate species were retrieved from the GenBank database. Multiple sequence alignment was performed using MUSCLE (version 3.8.31) with default parameters, and poorly aligned regions were trimmed to ensure phylogenetic reliability. A phylogenetic tree was then constructed using the neighbor-joining method implemented in MEGA12 software (version 12.1.1), with the Poisson model selected for amino acid substitution and 1000 bootstrap replicates performed to assess the statistical confidence of each node.

### 2.8. Statistical Analysis

Biochemical experiments were replicated at least three times. Data of APN expression are expressed as mean ± SD. Statistical analyses were performed by one-way ANOVA test using GraphPad Prism Software version 8.00. The *p* value < 0.05 was considered statistically significant.

## 3. Results

### 3.1. Influence of Trypsin on PDCoV Replication in Cell Lines of Distinct Host Origins

**Figure 1:**
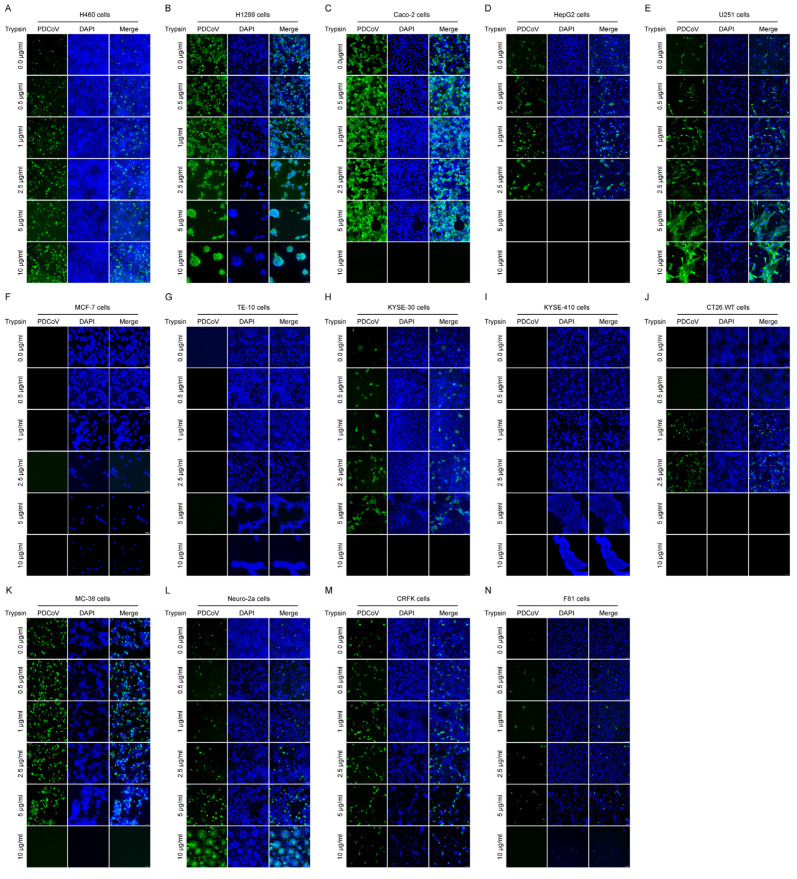
Effects of trypsin on PDCoV replication in cell lines from distinct host origins. Cells from human, murine, and feline were seeded into 24-well plates and cultured in a humidified atmosphere at 37 °C with 5% CO_2_. When the cells reached 100% confluency, the cells were infected with PDCoV at an MOI of 0.1. After 1 h of incubation, the cells were washed with PBS for two times to remove unbound viruses. Subsequently, 1 mL of DMEM with serial concentrations of trypsin (0, 0.5, 1, 2.5, 5, and 10 μg/mL) was added to the plates and incubated at 37 °C. At 24 h post-infection, cells were fixed and subjected to IFA to detect PDCoV NP protein (green), with nuclei stained with DAPI (blue). Representative images are shown for each cell line: (**A**) H460 cells, (**B**) H1299 cells, (**C**) Caco-2 cells, (**D**) HepG2 cells, (**E**) U251 cells, (**F**) MCF-7 cells, (**G**) TE-10 cells, (**H**) KYSE-30 cells, (**I**) KYSE-410 cells, (**J**) CT26.WT cells, (**K**) MC-38 cells, (**L**) Neuro-2a cells, (**M**) CRFK cells, and (**N**) F81 cells. Scale bar, 100 μm.

To evaluate the effects of trypsin on PDCoV replication in diverse host cell lines, we performed IFA to analyze PDCoV replication in 14 distinct cell lines incubated with gradient of trypsin concentrations (0, 0.5, 1, 2.5, 5, and 10 μg/mL). As illustrated in Figure 1, trypsin exerted different modulatory effects on PDCoV replication in different cell lines. Notably, human H1299, Caco-2, and MC-38 cells displayed high susceptibility to PDCoV, sustaining robust viral replication even in the absence of exogenous trypsin supplementation (Figure 1B,C,K). Moreover, trypsin has marginal effects on PDCoV replication efficacy in H1299, Caco-2, and MC-38 cells, as evidenced by invariable immunofluorescence intensity with increasing trypsin concentrations (Figure 1B,C,K). However, high concentrations of trypsin triggered prominent syncytium formation in PDCoV-infected H1299 cells (Figure 1B). H1299, Caco-2 and MC-38 cells tolerated trypsin at maximum concentrations of 2.5, 5 and 5 μg/mL, respectively, and higher trypsin levels triggered cell detachment from culture plates (Figure 1B,C,K). In semi-permissive cell lines (e.g., H460, U251, Neuro-2a), PDCoV replication was enhanced by trypsin in a concentration-dependent manner (Figure 1A,E,L). Obvious syncytia were visible in U251 and Neuro-2a cells under a high concentration of trypsin (Figure 1E,L). PDCoV could replicate in HepG2, KYSE-30, and CT26.WT, CRFK, and F81, whereas the addition of exogenous trypsin could increase viral yields (Figure 1D,H,J,M,N). HepG2, CT26.WT, CRFK, and F81 cells endured trypsin concentrations of a maximum of 2.5 μg/mL, while higher concentrations triggered cytotoxicity and cell detachment (Figure 1D,J,M,N). By comparison, KYSE-30 cells tolerated trypsin up to 5 μg/mL (Figure 1H). Three cell lines, namely MCF-7, TE-10, and KYSE-410, exhibited complete non-permissiveness to PDCoV. No fluorescent signals could be visualized regardless of supplementation with the highest trypsin concentrations used in this study (Figure 1F,G,I).

### 3.2. Human Lung and Colorectal Cells Are Highly Permissive for PDCoV Infection

**Figure 2:**
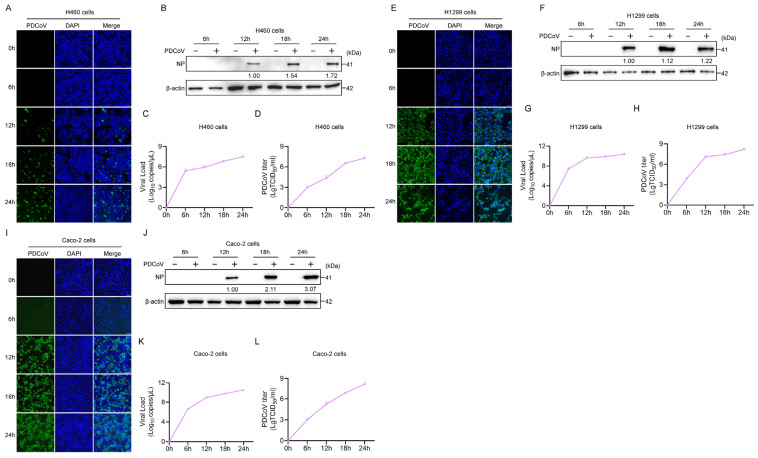
Susceptibility of human lung and colorectal cells to PDCoV infection. H460 (**A**–**D**), H1299 (**E**–**H**), and Caco-2 (**I**–**L**) cells were seeded in 24-well plates and maintained in a humidified 37 °C incubator with 5% CO_2_. At 100% confluency, cells were inoculated with PDCoV at an MOI of 0.1. Following 1 h of adsorption at 4 °C, cells were washed twice with PBS to remove unbound virus, then overlaid with 1 mL of DMEM supplemented with 2 μg/mL trypsin and incubated at 37 °C. At the indicated time points post-infection, cells and/or culture supernatants were harvested for IFA, Western blotting, RT-qPCR, and TCID_50_ assay to characterize the replication kinetics of PDCoV. Scale bar, 100 μm.

The above results showed that most of the cell lines could tolerate up to 2.5 μg/mL trypsin, so the subsequent experiments were performed with 2 μg/mL trypsin. CoVs mainly induce diseases of the respiratory and gastrointestinal tracts. Here, we evaluated the susceptibility of human lung (H460 and H1299) and colorectal (Caco-2) cells to PDCoV. To characterize the replication kinetics of PDCoV in human lung and colorectal cell lines, we infected H460, H1299, and Caco-2 cells with PDCoV at a multiplicity of infection (MOI) of 0.1, and monitored viral replication at sequential time points post-infection (0, 6, 12, 18, and 24 h). IFA results revealed a time-dependent increase in PDCoV NP protein expression in these three cell lines, with detectable NP signal emerging at 12 hpi and progressively intensifying through 24 h (Figure 2A,E,I). Consistent with IFA results, Western blotting further confirmed the accumulation of NP protein during PDCoV infection in H460 (Figure 2B), H1299 (Figure 2F), and Caco-2 cells (Figure 2J). To characterize the proliferation properties of PDCoV in these three cell lines, RT-qPCR and TCID_50_ assay were performed. As shown in Figure 2C,D,G,H,K,L, PDCoV productively replicates in H460, H1299, and Caco-2 cells, and reached the highest infectious titer at 24 hpi.

### 3.3. Human Cells Originating from Different Organs Display Heterogeneous Susceptibility to PDCoV

**Figure 3:**
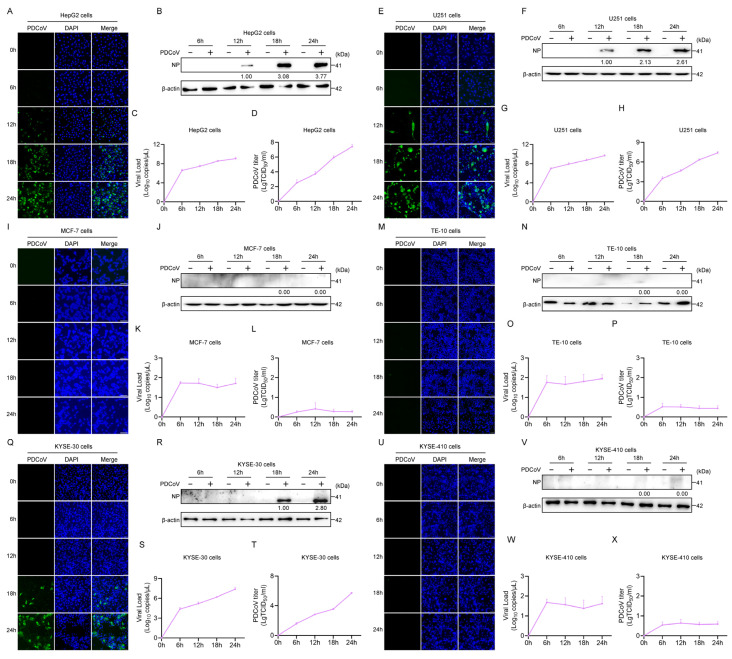
Susceptibility of human cells originating from different organs to PDCoV infection. HepG2 (**A**–**D**), U251 (**E**–**H**), MCF-7 (**I**–**L**), TE-10 (**M**–**P**), KYSE-30 (**Q**–**T**), and KYSE-410 (**U**–**X**) cells were seeded in 24-well plates and maintained in a humidified 37 °C incubator with 5% CO_2_. At 100% confluency, cells were inoculated with PDCoV at an MOI of 0.1. Following 1 h of adsorption, cells were washed twice with PBS to remove unbound virus, then overlaid with 1 mL of DMEM supplemented with 2 μg/mL trypsin and incubated at 37 °C. At the indicated time points post-infection, cells and/or culture supernatants were harvested for IFA, Western blotting, RT-qPCR, and TCID_50_ assay to characterize the replication kinetics of PDCoV. Scale bar, 100 μm.

To investigate the permissiveness of human cells originating from different organs to PDCoV infection, we evaluated viral replication kinetics in multiple cell types representing hepatic (HepG2), neural (U251), mammary (MCF-7), esophageal (TE-10, KYSE-30, KYSE-410) origins. Western blotting and IFA results revealed that PDCoV NP protein was detectable at 12 hpi in HepG2 and U251 cells, with signal intensity progressively increasing up to 24 hpi, indicative of productive viral replication (Figure 3A,B,E,F). Conversely, PDCoV NP expression was undetectable in MCF-7, TE-10 and KYSE-410 cells at every time point examined by IFA and Western blotting (Figure 3I,J,M,N,U,V). In KYSE-30 cells, PDCoV NP was detectable starting at 18 hpi, and its expression gradually increased through 24 hpi (Figure 3Q,R). Viral load quantification by RT-qPCR and TCID_50_ titration demonstrated exponential viral growth in HepG2 (Figure 3C,D) and U251 (Figure 3G,H) with viral loads reaching about 10^9^ copies/μL and infectious titers peaking at about 10^7^ TCID_50_/mL by 24 hpi. In KYSE-30 cells, the viral load reached about 10^7^ copies/μL and 10^5^ TCID_50_/mL by 24 hpi (Figure 3S,T), while MCF-7 (Figure 3K,L), TE-10 (Figure 3O,P), and KYSE-410 (Figure 3W,X) cells showed no increase in viral load or infectious titer over 24 hpi, confirming their non-permissive phenotype. These data revealed that PDCoV displays a selective tropism for human hepatic (HepG2), neural (U251), and esophageal (KYSE-30) cells, whereas mammary (MCF-7) and esophageal (TE-10, KYSE-410) cell lines are completely non-permissive.

### 3.4. PDCoV Productively Replicates in Multiple Murine Cell Lines

**Figure 4:**
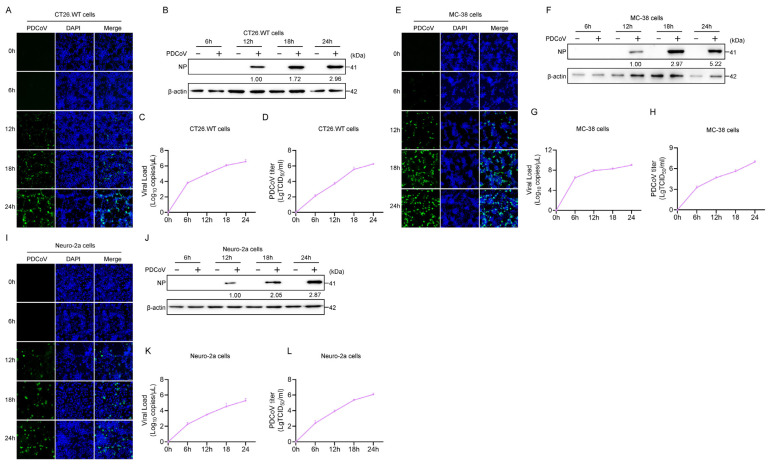
Susceptibility of murine cell lines CT26.WT, MC-38, and Neuro-2a to PDCoV infection. CT26.WT (**A**–**D**), MC-38 (**E**–**H**), Neuro-2a (**I**–**L**) cells were seeded in 24-well plates and maintained in a humidified 37 °C incubator with 5% CO_2_. At 100% confluency, cells were inoculated with PDCoV at an MOI of 0.1. Following 1 h of adsorption, cells were washed twice with PBS to remove unbound virus, then overlaid with 1 mL of DMEM supplemented with 2 μg/mL trypsin and incubated at 37 °C. At the indicated time points post-infection, samples were harvested for IFA, Western blotting, RT-qPCR, and TCID_50_ assay to characterize the replication kinetics of PDCoV. Scale bar, 100 μm.

Previous studies have reported that PDCoV can experimentally infect mice in vivo [18,38]. To assess the replication kinetics of PDCoV in murine cells, we infected CT26.WT, MC-38, and Neuro-2a cell lines and monitored viral replication over 24 h. IFA and Western blotting analysis revealed detectable NP signal emerged at 12 hpi in all three cell lines, with progressively intensified expression through 24 hpi (Figure 4A,B,E,F,I,J). Correspondingly, RT-qPCR and TCID_50_ assays demonstrated that viral RNA load and infectious viral titers rose steadily in all cell lines, peaking at 24 hpi, confirming productive PDCoV replication (Figure 4C,D,G,H,K,L). Collectively, these data establish that PDCoV efficiently infects and replicates in CT26.WT, MC-38, and Neuro-2a cells.

### 3.5. Feline CRFK and F81 Cells Are Permissive for PDCoV Infection

**Figure 5:**
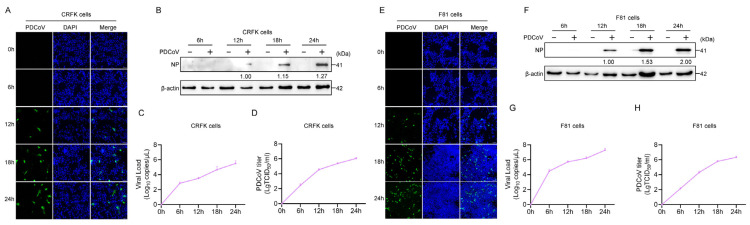
Susceptibility of feline cell lines CRFK and F81 to PDCoV infection. CRFK (**A**–**D**) and F81 (**E**–**H**) cells were seeded in 24-well plates and maintained in a humidified 37 °C incubator with 5% CO_2_. At 100% confluency, cells were inoculated with PDCoV at an MOI of 0.1. Following 1 h of adsorption, cells were washed twice with PBS to remove unbound virus, then overlaid with 1 mL of DMEM supplemented with 2 μg/mL trypsin and incubated at 37 °C. At the indicated time points post-infection, samples were harvested for IFA, Western blotting, RT-qPCR, and TCID_50_ assay to characterize the replication kinetics of PDCoV. Scale bar, 100 μm.

To evaluate the risk of companion animals, such as cats, acting as reservoir hosts and intermediate transmission vehicles for PDCoV, we characterized the replication kinetics of PDCoV in feline lines, the CRFK and F81 cell lines. CRFK and F81 cells were inoculated with PDCoV at an MOI of 0.1, and cell samples were harvested at 0, 6, 12, 18, and 24 hpi. In CRFK cells, IFA results revealed that PDCoV NP protein became detectable at 12 hpi, with progressively intensified expression through 24 hpi, indicative of productive viral infection (Figure 5A). Western blotting analysis confirmed time-dependent accumulation of NP protein, increasing from 1.00 at 12 hpi to 1.15 at 18 hpi and 1.27 at 24 hpi (Figure 5B). Correspondingly, RT-qPCR and TCID_50_ assays demonstrated that viral RNA load and infectious viral titer rose steadily over time, peaking at 24 hpi, confirming robust PDCoV replication in CRFK cells (Figure 5C,D). In F81 cells, immunofluorescence signal of NP was detectable at 18 hpi and intensifying through 24 hpi (Figure 5E). Western blotting analysis revealed gradual NP accumulation, with normalized band intensities reaching 1.00 at 18 hpi and 1.18 at 24 hpi (Figure 5F). Consistent with these findings, viral RNA load (Figure 5G) and infectious titer (Figure 5H) in F81 cells increased in a time-dependent manner, peaking at 24 hpi, demonstrating efficient PDCoV replication in F81 cells. These results demonstrate that PDCoV can productively infect and replicate in two feline cell lines (CRFK and F81), as evidenced by time-dependent increases in viral protein expression, RNA load, and infectious viral titers.

### 3.6. Relative Expression Profile of APN in Cell Lines of Distinct Host Origins

**Figure 6:**
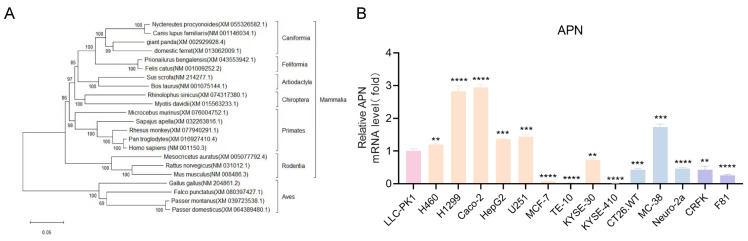
Phylogenetic and expression profile of APN in cell lines of distinct host origins. (**A**) Phylogenetic analysis of APN nucleotide sequences from representative vertebrate species. The tree was constructed using the neighbor-joining method with 1000 bootstrap replicates, and bootstrap values are labeled at internal nodes. The scale bar indicates 0.05 nucleotide substitutions per site. (**B**) Relative APN mRNA expression levels in a panel of mammalian and avian cell lines. Expression was normalized to LLC-PK1 cells (set as 1.0). Data are presented as mean ± SD from at least three independent experiments. Statistical significance relative to LLC-PK1 cells was analyzed by one-way ANOVA test: ** *p* < 0.01; *** *p* < 0.001; **** *p*< 0.0001.

APN plays an important role in PDCoV replication [39], phylogenetic analysis of the *APN* gene across representative vertebrates revealed a well-resolved topology with a basal split separating avian and mammalian clades (100% bootstrap support), where mammalian lineages formed six robust monophyletic orders (*Rodentia*, *Primates*, *Chiroptera*, *Artiodactyla*, *Feliformia*, and *Carnivora*) largely congruent with established taxonomy, providing robust evolutionary insights into the *APN* gene across diverse vertebrate lineages (Figure 6A).

To explore the potential correlation between *APN* expression and PDCoV permissiveness, we quantified the relative mRNA levels of APN in these cell lines using RT-qPCR. Compared with LLC-PK1 cells, H1299 and Caco-2 cells exhibited higher APN mRNA levels, while other cell lines (KYSE-30, Neuro-2a, CT26.WT, CRFK and F81) displayed lower expression levels (Figure 6B). Notably, the cell lines (e.g., H1299, Caco-2, MC-38, U251, and Neuro-2a) identified as highly permissive for PDCoV replication demonstrated higher APN expression (Figure 1), whereas non-permissive cell lines (e.g., MCF-7, TE-10, KYSE-410) showed minimal APN transcript levels (Figure 6B). These results suggest a potential link between basal APN expression and the cellular susceptibility to PDCoV infection.

## 4. Discussion

CoVs form a large viral family that infects mammals and birds, causing a diverse range of diseases in these hosts. For a virus to achieve cross-species infection, a fundamental prerequisite is its ability to replicate successfully in cells derived from various host species. For instance, a study has found that at least seven cell lines originating from Thomas’s horseshoe bats, king horseshoe bats, humans, palm civets, African green monkeys, and ferrets are all highly susceptible to SARS-CoV-2 [40]. Phylogenetic and evolutionary analyses have revealed a close genetic relationship between PDCoV and sparrow coronavirus HKU17 (SpCoV HKU17), which suggests that PDCoV may have undergone cross-species transmission from avian species to mammals [41].

Previous studies have documented that PDCoV can infect different organs in PDCoV-challenged pigs, including duodenum, jejunum, ileum, cecum, colon, liver, spleen, kidney, lung, and mesenteric lymph nodes (MLN) [42,43]. Although previous studies have confirmed that PDCoV is capable of replicating in several cell lines including A549, iPAM, Vero, MDBK, DF1, bovine mesenchymal, and Huh7 cells [44,45,46], the current understanding of the susceptibility of different cell lines to PDCoV remains far from comprehensive. In this study, Human cell lines were prioritized because recent evidence of PDCoV RNA in pediatric patients with acute febrile illness in Haiti raised concerns about human susceptibility [19]. To assess the breadth of tissue tropism, we selected cell lines derived from multiple organ systems, including respiratory, digestive, hepatic, nervous, and mammary tissues. Previous in vivo studies had already demonstrated that mice are experimentally susceptible to PDCoV [18,47], so we also choose well-characterized murine cell lines (CT26.WT, MC 38, Neuro 2a) for evaluation of its susceptibility, with the goal of informing the selection of appropriate mouse models for future in vivo investigations. Furthermore, companion animals such as cats may serve as viral reservoirs or intermediate hosts, particularly since feline species are permissive to other coronaviruses and their frequent close contact with humans could facilitate cross-species transmission. So, this study employed feline cell lines (CRFK, F81) to evaluate their susceptibility to PDCoV.

Exogenous trypsin is a key factor regulating the in vitro replication of many CoVs, and its core mechanism lies in the proteolytic cleavage of the viral S protein at the S1/S2 site, which triggers conformational changes in the S protein, mediates viral membrane-host cell membrane fusion, and initiates viral entry into host cells [48]. In this study, we found that trypsin exerts a cell-type-specific modulatory effect on PDCoV replication. For highly permissive cell lines (H1299, Caco-2, MC-38), PDCoV can achieve robust replication even without exogenous trypsin supplementation, and the replication level remains unchanged with the increase in trypsin concentration. The reason might be that these cell lines endogenously express sufficient proteases to complete the cleavage of the PDCoV S protein, making exogenous trypsin redundant for viral entry and replication. Notably, high concentrations of trypsin induced obvious syncytium formation in PDCoV-infected H1299 cells, which is consistent with the role of trypsin in promoting coronavirus cell–cell fusion [49].

PDCoV was originally identified as a swine enteric coronavirus, but subsequent studies have confirmed its experimental infection in multiple vertebrate species (wild birds, chickens, turkeys, calves, mice), and even PDCoV nucleic acid was detected in the plasma of pediatric patients with acute febrile illness, providing evidence for its potential human infection [19]. In this study, we found that PDCoV can efficiently replicate in multiple human cell lines derived from different organs, including lung (H1299, H460), colorectal (Caco-2), hepatic (HepG2), neural (U251), and esophageal (KYSE-30) cells. Among them, H1299 (lung origin) and Caco-2 cells (colorectal origin) supported the highest level of PDCoV replication, which is consistent with the characteristic of CoVs causing severe respiratory and enteric disease. In addition, PDCoV was also found to replicate productively in murine (CT26.WT, MC-38, and Neuro-2a), which is consistent with the results that mouse is a good model for in vivo experimental infection [18]. A recent study by Meng et al. demonstrated that cats are not susceptible to PDCoV, which contrasts with our results that feline cells are permissive to viral infection [14]. Actually, PDCoV can replicate in cultured cat cells due to the available APN receptor and added trypsin. In living cats, multiple host barriers, including weak receptor binding, lack of activating proteases, and strong antiviral immunity, stop successful infection.

APN is ubiquitously expressed across a broad range of tissues and cell types, and is well established as a specific functional receptor for human coronavirus 229E [50], canine coronavirus [51], feline infectious peritonitis virus, and transmissible gastroenteritis virus (TGEV) [52]. APN also plays an important role in PDCoV replication [53]. Our quantitative detection of APN mRNA expression in different cell lines further revealed a potential positive correlation between APN expression level and cellular susceptibility to PDCoV. The results further verify the core role of APN in PDCoV infection, and suggest that the basal expression level of APN is an important determinant of whether host cells can be infected by PDCoV. In addition, this study only detected the mRNA level of APN, and the protein expression level and subcellular localization of APN, which directly affect viral binding and entry, need to be further verified.

In conclusion, this study demonstrates that trypsin exerts cell-type-specific regulatory effects on PDCoV replication. Human cells (H1299, Caco-2) and murine MC-38 are highly susceptible to PDCoV infection, whereas MCF-7, TE-10, and KYSE-410 are completely non-permissive. This study also reveals a potential correlation between APN expression and cellular susceptibility to PDCoV. It is urgent to employ in vivo animal models to evaluate the transmissibility of PDCoV and to investigate its transmission dynamics.

## Data Availability

The raw data supporting the conclusions of this article will be made available by the authors on request.

## Supplementary Materials

The following supporting information can be downloaded at: https://www.mdpi.com/article/10.3390/v18070776/s1, Table S1: Primer sequences used in this study.
