# Supplementary material for: Susceptibility of Cell Lines from Different Species to Porcine Deltacoronavirus
Source: Viruses. 2026 Jul 15;18(7):776. doi: 10.3390/v18070776 (PMC13431601; doi:10.3390/v18070776)
Supplement: Supplementary file 1 [file viruses-18-00776-s001.zip › viruses-4423987-supplementary.pdf]

Table S1: Primer sequences used in this study

Gene ID	Sequence (5'-3')
qPCR-felineAPNF	TCAGCGAGGTGTTTGACTCC
qPCR-felineAPNR	TTGATGGTCGGCTGTTTGTC
qPCR-humanAPNF	GACATTGACAAGACTGAGCTG
qPCR-humanAPNR	CTGCATCTGTGTAGTGGCCA
qPCR-murineAPNF	TACCTGACTCCTACCGGGTG
qPCR-murineAPNR	GAGTGCCGTCCAGGGTTCGC
qPCR-porcineAPNF	CACACCTGTGATGTCCACGT
qPCR-porcineAPNR	GGGGAGTGGGTAGGGTGTAT
qPCR-felineactinF	AGAAGCTGTGCTACGTGGC
qPCR-felineactinR	CCAGGAAGGAAGGCTGGAAG
qPCR-murineactinF	CTGGTCGTACCACAGGCATT
qPCR-murineactinR	GGTAGTCTGTCAGGTCCCGG
qPCR-hactinF	GATCTGGCACCACACCTTCT
qPCR-hactinR	GGGGTGTTGAAGGTCTCAAA
qPCR-PDCoV-NF	CCCAGCTCAAGGTTTCAGAG
qPCR-PDCoV-NR	ATTGGCACCAGTACGAGACC
